# P87 Artificial intelligence in antimicrobial resistance surveillance: comparative analysis of global dashboards and predictive metrics

**DOI:** 10.1093/jacamr/dlag102.093

**Published:** 2026-06-26

**Authors:** Aser Waleed Mohamed Marzok, Rasha Abdelsalam Elshenawy

**Affiliations:** School of Health, Medicine and Life Sciences, University of Hertfordshire, College Lane, Hatfield, AL10 9AB, UK; School of Health, Medicine and Life Sciences, University of Hertfordshire, College Lane, Hatfield, AL10 9AB, UK

## Abstract

**Background:**

Antimicrobial resistance poses an escalating global public health threat, with carbapenem-resistant *Klebsiella pneumoniae* rising 61% above European Union baselines and only 39% of National Action Plans fully financed.^1^. Major health agencies, such as the WHO, the ECDC, the UK Health Security Agency (UKHSA), the United States CDC, Africa CDC, the Australian Antimicrobial Resistance Network (AAMRNet), and Public Health Ontario (PHO) have developed surveillance systems and dashboards to monitor resistance trends and antimicrobial consumption.^2^ Artificial intelligence offers transformative potential for real-time, predictive surveillance of antimicrobial resistance, presenting a timely opportunity to evaluate how existing frameworks and data infrastructures can be optimized for AI integration.^3^

**Objectives:**

To evaluate how antimicrobial resistance surveillance dashboards and metric frameworks across WHO, ECDC, UKHSA, US CDC, Africa CDC, PHO, and AAMRNet, and how AI could be used to enable decision-making, and to identify policy gaps limiting predictive, actionable surveillance.

**Methods:**

A comparative policy analysis was conducted across seven global AMR surveillance systems using publicly available reports (2022–2025). Frameworks were examined across five domains: dashboard purpose, reported metrics, data standardization, interoperability, and policy translation. AI readiness was assessed using predefined criteria, including data structure, timeliness, integration of microbiology and antimicrobial use datasets, and predictive analytics capacity. Data extraction followed a structured framework, and findings were analysed using quantitative benchmarking and qualitative thematic analysis. Ethical approval was not required, as only publicly available aggregate data were used.

**Results:**

Quantitative analysis across five domains demonstrated considerable variation in surveillance maturity, with ECDC, UKHSA, and US CDC emerging as the most advanced systems, offering robust dashboard infrastructure, standardized resistance metrics, and strong interoperability. Resistance data confirmed meaningful progress, with MRSA bloodstream infections declining 20.4% below 2019 EU baselines, representing the first EU target successfully achieved. Laboratory participation increased substantially, with 1993 laboratories reporting to ECDC in 2024. Qualitative thematic analysis identified four constructive themes: strengthening surveillance infrastructure, expanding real-time monitoring capabilities, advancing antimicrobial stewardship programmes, and progressing One Health integration. Canada's health equity framework and the WHO Antimicrobial Resistance Accountability Index represent emerging best-practice models. Regarding artificial intelligence readiness, existing surveillance frameworks provide promising foundational datasets. Structured data pipelines, harmonized resistance metrics, and integrated antimicrobial consumption data, already present in leading systems, represent valuable assets upon which predictive modelling, real-time genomic surveillance, and AI-enabled decision-making tools can be systematically developed and scaled across all seven agencies reviewed.

**Conclusions:**

Global antimicrobial resistance surveillance systems are strengthening, with leading agencies demonstrating robust infrastructure, expanding dashboards, and improving stewardship outcomes. These frameworks provide a strong foundation for AI integration, particularly where standardized, interoperable, longitudinal data exist. Enhancing monitoring of antibiotic utilization and enabling effective stewardship implementation are critical to improving patient outcomes and saving lives. However, financing gaps, retrospective reporting limitations, and One Health integration require attention. Prioritizing real-time genomic surveillance, harmonized data pipelines, and AI-enabled predictive modelling can transform surveillance into actionable, anticipatory public health intelligence.
